# New insights into RNA mycoviruses of fungal pathogens causing Fusarium head blight

**DOI:** 10.1016/j.virusres.2024.199462

**Published:** 2024-09-13

**Authors:** Živilė Buivydaitė, Anne Winding, Lise Nistrup Jørgensen, Athanasios Zervas, Rumakanta Sapkota

**Affiliations:** a Department of Environmental Science, Aarhus University, Frederiksborgvej 399, Roskilde 4000, Denmark; b Department of Agroecology, Aarhus University, Forsøgsvej 1, Slagelse 4200, Denmark

**Keywords:** Virome, Transcriptomics, Wheat, FHB, Mycovirology

## Abstract

Fusarium head blight (FHB) continues to be a major problem in wheat production and is considered a disease complex caused by several fungal pathogens including *Fusarium culmorum, F. graminearum* and *F. equiseti*. With the objective of investigating diversity of mycoviruses in FHB-associated pathogens, we isolated *Fusarium* spp. from six wheat (*Triticum aestivum*) cultivars. In total, 56 *Fusarium* isolates (29 *F. culmorum*, 24 *F. graminearum*, one *F. equiseti*) were screened for mycoviruses by extracting and sequencing double-stranded RNA. We found that a large proportion of *Fusarium* isolates (46 %) were infected with mycoviruses. *F. culmorum*, previously described to harbor only one mycovirus, tended to host more viruses than *F. graminearum*, with a few isolates harboring seven mycoviruses simultaneously. Based on the RNA-dependent RNA polymerase domain analysis, ten were positive-sense single-stranded RNA viruses (related to viruses from families *Mitoviridae, Botourmiaviridae, Narnaviridae, Tymoviridae, Gammaflexiviridae*, as well as proposed Ambiguiviridae and ormycovirus viral group), one was double-stranded RNA virus (*Partitiviridae*), and five were negative-sense single-stranded RNA viruses (related to members in the families of *Yueviridae, Phenuiviridae, Mymonaviridae*, as well as proposed Mycoaspiviridae). Five mycoviruses were shared between *F. graminearum* and *F. culmorum*. These results increase our general understanding of mycovirology. To our knowledge, this is the first in-depth report of the mycovirome in *F. culmorum* and the first report on the diversity of mycoviruses from Danish isolates of FHB-causing fungi in general.

## Highlights

• Many fusarium head blight associated isolates were infected with mycoviruses.
• *Fusarium culmorum* hosted more mycoviruses than F*usarium graminearum*.
• 10 positive, 5 negative-sense and 1 double-stranded RNA mycovirus was detected.

## 1 Introduction

Wheat (*Triticum aestivum*) is a major source of carbohydrates for both humans and livestock, and it is the most widely cultivated grain in the European Union (FAO, 2022). However, crop diseases, such as Fusarium head blight (FHB), pose a threat to wheat production. FHB is a fungal disease that not only affects wheat, but also other small-grain cereals and maize. It frequently leads to reduced grain quality due to mycotoxin contamination and decreased crop yield. FHB is caused by plant pathogenic *Fusarium* species complex, with *Fusarium graminearum* and *F. culmorum* being among the most prevalent species in Europe (Nielsen et al., 2011; Wagacha and Muthomi, 2007).

Mycoviruses, which are viruses that infect fungi, have been detected across all major fungal taxa (Ayllón and Vainio, 2023). While mycoviruses typically contain RNA genomes and have an intracellular lifestyle, as well as exert little impact on their hosts, exceptions to these traits have been observed too (Kotta-Loizou, 2021). The potential application of mycoviruses in the biocontrol of phytopathogenic fungi, such as *Fusarium*, has propelled the field of mycovirology (García-Pedrajas et al., 2019). Moreover, the advancements seen in high-throughput sequencing technologies in recent years have enabled researchers to discover a multitude of novel mycoviruses. Given the economic importance of *Fusarium* as a pathogen, its mycoviruses have been studied quite extensively compared to other fungi. Over 30 fully sequenced mycoviruses have been reported in at least 14 different species of *Fusarium* (Li et al., 2019; Mahillon et al., 2020). Furthermore, several of them have been shown to induce reduced virulence (hypovirulence) in *F. graminearum* (Chu et al., 2002; Li et al., 2019, 2016, 2020; Paudel et al., 2022). Conversely, *F. culmorum* has only one mycovirus described so far (Mahillon et al., 2020), though several double-stranded (ds) RNAs indicative of viral infection were reported (Tóth et al., 2005). Although *Fusarium* is among the most widely studied fungal pathogens, knowledge about its viral diversity and its impacts on the disease development is limited.

In this study we aim to gain new insights into the viruses of the FHB species complex, thereby contributing to the broader understanding of mycovirology. To achieve this, we sequenced dsRNA from 54 isolates of *Fusarium culmorum, F. graminearum* and *F. equiseti*. Furthermore, anecdotal discussions suggest that mycoviruses can often be lost through continuous culturing or storage of fungal isolates in culture collections over prolonged periods. To avoid artificially lower mycovirus diversity, we chose to isolate *Fusarium* spp. directly from wheat fields in Denmark, rather than using previously isolated *Fusarium* strains available from culture collections. Here we present previously identified as well as novel mycoviruses found in these *Fusarium* spp. Additionally, we analyze sequence characteristics, genome structures and perform phylogenetic analyses of identified mycoviruses.

## 2 Methods

### 2.1 Wheat sampling

Wheat heads with obvious FHB symptoms were harvested in July 2021 and 2022 from an experimental field trial at the Flakkebjerg research station, Aarhus University, Denmark, where different wheat cultivars were screened for resistance against FHB (Jørgensen et al., 2023). Wheat plots consisting of six different wheat cultivars (Sheriff, Rembrandt, Hereford, Torp, Sauvignon and Oakley) were artificially infected with a mix of deoxynivalenol producing *F. culmorum* and *F. graminearum* isolates. Artificial infection of wheat with *Fusarium* spp. was carried out via spore spray on the foliar canopy during flowering or by placing infected grains on the ground. In addition to sampling wheat heads from artificially infected wheat, we also collected wheat heads with naturally occurring FHB infection from nearby fields at the research station. Collected wheat heads were stored in separate paper bags for each field plot, at room temperature for approximately two months, before the isolation of *Fusarium* spp. took place.

### 2.2 *Fusarium* spp. isolation and identification

Wheat kernels were first surface-sterilized by 5 % sodium hypochlorite for 7 min while shaking, followed by rinsing three times with sterile Milli-Q water (Clear and Patrick, 2000). After the final rinse, the kernels were allowed to dry before being placed on potato dextrose agar (PDA) plates containing 100 mg/L streptomycin (Sigma-Aldrich). Plates were kept under black light for a period of 3 to 7 days. Isolates were then selected using two strategies – either by isolating single conidia or subculturing hyphae.

For single conidia isolation, spore suspension was prepared by flooding the PDA plates with sterile water and agitating. A drop of the suspension was then evenly distributed to a new plate containing water agar. Light microscope (Zenith Microlab 1000, magnification 100x) was used to locate a single macroconidium inside the microscope field. The agar spot was marked using a modified La Rue cutter, and the piece of agar was then transferred to a new PDA plate overlaid with filter paper (Whatman, grade 1) under a dissection microscope. The isolates were preserved as mycelium plugs kept in a solution of 20 % glycerol at a temperature of −80 °C. If the developing fungal colonies were not producing spores, they were instead transferred to a synthetic nutrient-poor agar (SNA) medium (Leslie and Summerell, 2006a) and subjected to the same procedure for isolating conidia.

To avoid the loss of mycoviruses during single conidia isolation, several isolates were obtained using an alternative hypha subculturing strategy. Here, small amounts of hyphae were excised from colonies emerging from the kernels and were transferred to new PDA plates overlaid with filter paper. Only isolates with homogeneous growth were selected.

To identify *Fusarium* species, small amounts of hyphae were mixed with 100 μl of nuclease-free water and heated at 95 °C for 10 min. After centrifugation for 2 min at 10,000 × *g*, 5 μl of supernatant was used in a polymerase chain reaction (PCR) to amplify the partial translation elongation factor 1α gene as described in Cobo- Díaz et al. (2019). The amplification products were sequenced using the Sanger method (Macrogen, Netherlands), the resulting sequences were trimmed using Geneious Prime software, and Fusarium species were identified using basic local alignment search tool (BLAST) against the FUSARIUM-ID v.3.0 database (Torres-Cruz et al., 2021) and FUSARIOID-ID database (www.fusarium.org) (Crous et al., 2021).

Information associated with each fungal isolate (fungal species, wheat cultivar from which *Fusarium* was isolated, isolation method, year, source of fungal infection, as well as wheat plot (number serving as a distinct identified encapsulating all associated variables)), is provided in Supplementary Table S1.

### 2.3 dsRNA extraction and library preparation

From a total of 112 isolates, three to four isolates from each wheat plot were randomly chosen for dsRNA extraction, resulting in a final set of 54 isolates that were sequenced individually. Approximately 100 mg of mycelium were collected after 7 days of growing on the PDA plates overlaid with filter paper. Before extraction, the mycelium was stored in tubes containing 400 mg of 0.5 mm glass beads at −80 °C. Total RNA was extracted using 800 μl of PRImeZOL Reagent (Canvax Biotech, Spain) by incubating the mixture for 10 min and then homogenizing using a bead mill homogenizer (Bead Ruptor Elite, Omni International, USA) for two cycles of 30 s at 4 m *s*^−1^ speed. Subsequently, protocols by Okada et al. (2015) and Myers et al. (2020) were followed with minor modifications. After centrifugating the mixture at 14,000 × *g* for 10 min at 4 °C, 160 μl of chloroform was added to the supernatant. The mixture was then vortexed for 1 min, incubated for 3 min, and centrifugated again at 3000  ×  g at 4 °C for 30 min. The aqueous phase containing total RNA was mixed with ethanol (final concentration 16 %) and loaded to a pre-prepared microcolumn used to separate dsRNA. The columns were prepared by piercing a hole at the bottom of 0.5 ml tube with a 19.5-gauge needle, adding 90 mg of cellulose D powder (Advantec), placing this column into a 2 ml Eppendorf tube, and shortly before use equilibrating it with 400 μl of sodium chloride-Tris-EDTA (STE) buffer (G-Biosciences) containing 16 % ethanol. After loading total RNA to a column, the column was washed three times with 400 μl of STE buffer containing 16 % ethanol. The dsRNA was eluted with 400 μl STE buffer and precipitated by adding 0.1 vol. of 3 M sodium acetate and 3.5 vol. of 96 % ethanol and incubated at −20 °C overnight. Then, the tubes were centrifuged at 18,000 g for 10 min to remove supernatant. The pellet was washed twice with 75 % ethanol followed by identical centrifugation steps, and left to air-dry for approximately 30 min. The precipitated dsRNA samples were then dissolved in 20 µL of 2 x STE and treated with 2 U of DNase I, RNase-free, and 50 U of S1 Nuclease (Thermo Scientific, Lithuania). The samples were precipitated again as described above, to remove the enzymes, and eluted in STE buffer. RNA concentration was then measured using the Qubit RNA High Sensitivity assay on a Qubit 4 Fluorometer (Invitrogen, Oregon, USA) and visualized on a 4150 TapeStation (Agilent Technologies) using RNA HS reagents.

The NEBNext Ultra II Directional RNA Library Prep Kit for Illumina (New England Biolabs) and the Multiplex Oligos for Illumina were used to prepare and multiplex the 54 sequencing libraries with the following modifications to the standard protocol: the extracted RNA was fragmented for 15 min at 94 °C and the first-strand synthesis reaction was conducted at 42 °C for 30 min. The libraries were pooled equimolarly and sequenced on an Illumina NextSeq 500 platform using the 300 cycles chemistry in pair-end mode (2 × 151 bp sequencing).

### 2.4 RT-PCR

The presence of several randomly selected viruses was verified by RT-PCR. Primers were designed using Primer3 (v. 2.3.7) within Geneious Prime (Dotmatics) (v. 2024.0.4). For the amplification, 5 μl of dsRNA were used in the cDNA synthesis using Transcriptor First Strand cDNA Synthesis Kit (Roche), employing random hexamer primers. Then, mycovirus-specific amplification reactions were performed consisting of 5 μl cDNA, 12.5 μl 2x PCRBIO Ultra Mix (PCR Biosystems Inc., United Kingdom), 0.5 μl bovine serum albumin and 1 μl of each forward and reverse primers (10 μM) on a SimpliAmp Thermal Cycler (Applied Bio-systems, Foster City, California, US), with a cycling program as follows: 95 °C for 3 min, 40 cycles of 95 °C for 30 s, 61 °C for 30 s, 72 °C for 30 s, and the final elongation at 72 °C for 5 min. The specific primers used for amplifications are detailed in Supplementary Table S2. The PCR products were analyzed by 2 % agarose gel electrophoresis (Supplementary Fig. S1.).

### 2.5 Bioinformatics

The quality of raw sequencing data was assessed using MultiQC (v.1.14) (Ewels et al., 2016). Reads shorter than 50 nt, adaptor sequences and low-quality bases (average Q-score <20 in a four-base window) were removed using Trimmomatic (v.0.39) (Bolger et al., 2014). The *de novo* co-assembly was performed on reads combined from all 54 samples using metaSpades (v.3.15.5) (Nurk et al., 2017), rnaviralSPAdes (v.3.15.5) (Meleshko et al., 2021) and Trinity (v2.12) (Grabherr et al., 2011). Three assembler algorithms were used to maximize the contig length. Contigs with a length shorter than 1000 nt were removed, and the remaining ones were mapped against nuclear and mitochondrial genomes of *F. culmorum* (GenBank GCA_016952355.1 and NC_026993.1) and *F. graminearum* (GCA_000240135.3 and NC_009493.1) using BBDuk (BBMap v. 38.18; https://sourceforge.net/projects/bbmap/). The unmapped contigs were screened for viral sequences using Hmmer (v.3.1b) (Eddy, 2011) against profile hidden Markov models of RdRp-scan (Charon et al., 2022) and RVMT databases (Neri et al., 2022). Contigs marked as viral were then dereplicated at 95 % nucleotide identity using dRep (v. 3.2.0; –S_algorithm ANImf -sa 0.95 -l 1000 –ignoreGenomeQuality –SkipMash) (Olm et al., 2017). The final virus list was further curated to remove transposon sequences and false positives. When chimeric assemblies were found, second-best assembly was selected based on dRep output. An exception was made for Fusarium culmorum botourmiavirus 1, where trimming was performed by aligning its sequence with the closest relatives (Genbank accession numbers OQ565623.1, ON812854.1 and OM514659.1). Additional non-RdRp-containing segments were found using Diamond BLASTx (v.2.0.14) (Buchfink et al., 2015) against custom reference database consisting of sequences of additional segments from the closest relatives of the viruses found here as well as phylogenetically related viruses.

Horizontal contig coverage in each sample was determined using CoverM (v. 0.6.1; https://github.com/wwood/CoverM) covered_fraction function (Supplementary Table S1). Mycovirus was considered present in a sample only if RdRp-coding contigs had a coverage >30 %. Heatmap was created using TBtools software (Chen et al., 2020) from mean coverage calculated by CoverM from reads normalized by random subsampling to the lowest sequencing depth (450,871 reads in each forward and reverse reads).

### 2.6 Genome characterization

Geneious Prime (Dotmatics) (v. 2024.0.4) was used to visualize viral metagenome-assembled genomes and find open reading frames (ORFs) via integrated ORF Finder from NCBI. Functional annotation was attempted using BLASTp, Motif search (against Pfam, NCBI-CDD, PROSITE pattern and profile databases; www.genome.jp/tools/motif/; only matches e-value below 0.01 are shown unless stated otherwise), HHpred (Zimmermann et al., 2018), Foldseek (van Kempen et al., 2023) and Phyre2 (Kelley et al., 2015).

RNA secondary structure of 3’ or 5’ untranslated regions (UTR) was predicted using the Mfold V2.3 server with folding temperature set at 25 °C (Zuker, 2003). The predicted structure was displayed using VARNAv3–93 (Ponty and Leclerc, 2015).

The amino acid (aa) sequences of RNA-dependent RNA polymerase (RdRp) were used to construct phylogenetic trees using the Maximum Likelihood method in IQ-TREE. Prior to this, sequences were aligned using multiple alignment using fast Fourier transform (MAFFT) E-INS-I algorithm (v7.310) (Katoh and Standley, 2013) and the best substitution model was determined using ModelFinder (Kalyaanamoorthy et al., 2017). Related sequences used in trees were obtained from BLASTp searches against NCBI nr database. The accession numbers of all the sequences used in the phylogenetic tree are available in Supplementary Table S3.

### 2.7 Data availability

All the raw sequencing reads were stored in the Sequence Read Archive (SRA) database with BioProject accession no PRJNA1125841.

## 3 Results

**Fig. 1:**
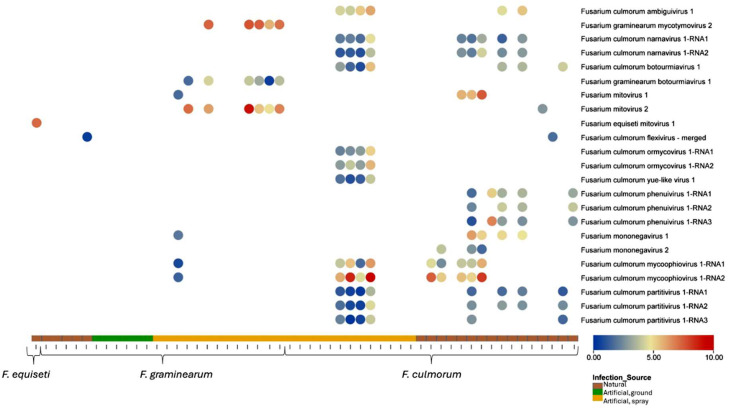
Heatmap displaying the relative abundance of mycoviruses in different *Fusarium* isolates. Bubble color represents log2 of mean coverage of each virus in reads normalized by subsampling to the lowest sequencing depth. X-axis indicates *Fusarium* isolates, and Y-axis represents viral contigs*.*

Among the 54 sequenced fungal isolates, 25 were found to host in total 16 different mycoviruses (Fig. 1). Specifically, 16 out of 29 (55 %) *F. culmorum* isolates were infected, with a maximum of seven viruses per isolate. Similarly, eight out of 24 (33 %) *F. graminearum* isolates were infected with mycoviruses, with each isolate carrying a maximum of three viruses per isolate. Only one *F. equiseti* isolate was obtained in our collection, and it was positive for one mycovirus. In total, eight unique viruses were found only in *F. culmorum*, one unique virus in *F. graminearum*, and five viruses were shared between *F. culmorum* and *F. graminearum*. Lastly, fungal isolates obtained from naturally occurring FHB-diseased field plots were more frequently infected with mycoviruses (63 %) compared to fungal isolates from artificially infected wheat (40 %).

Based on RdRp analysis, nine were positive sense (+) single-stranded (ss) RNA viruses belonging to the families *Narnaviridae, Botourmiaviridae, Mitoviridae, Gammaflexiviridae-*like and proposed Mycotymoviridae, Ambiguiviridae and ormycovirus virus group. Furthermore, five negative-sense (-) ssRNA mycoviruses were found (*Yueviridae*-like, *Phenuiviridae, Mymonaviridae*, as well as proposed Mycoaspiviridae), as well as one dsRNA virus (*Partitiviridae*). In total sixteen different mycoviruses were discovered, as summarized in Table 1.

**Table 1:** Summary of viruses discovered in this study.

Virus name (Abbreviation)	GenBank ID	Length (nt)	First BlastP hit of RdRp ORF	Iden. (% aa)	Cover (%)	Proposed taxonomic rank
Fusarium culmorum ambiguivirus 1 (FcAV1)	PP971521	5053	MAG:Riboviria sp. Second hit: Diaporthe ambigua RNA virus 1	72.3 / 56.2	87/87	*P_Kitrinoviricota;C_Tolucaviricetes;O_Tolivirales;F_"Ambiguiviridae"*
Fusarium graminearum mycotymovirus 2 (FgMTV2)	PQ000863	7886	Fusarium graminearum mycotymovirus 1, SX64	94.5	100	*P_Kitrinoviricota;C_Alsuviricetes;O_Tymovirales;F_Tymoviridae;G_"Mycotymovirus"*
Fusarium culmorum narnavirus 1 (FcNV1)	PQ000864	2991	Plasmopara viticola lesion associated orfanplasmovirus 1	50.5	97	*P_Lenarviricota;C_Miaviricetes;O_Ourlivirales;F_Narnaviridae*
	PQ000865	2629				
Fusarium culmorum botourmia virus 1 (FcBV1)	PQ000866	2601	Fusarium subglutinans ourmia-like virus isolate SJH 1–1	73.7	98	*P_Lenarviricota;C_Miaviricetes;O_Ourlivirales;F_Botourmiaviridae*
Fusarium graminearum botourmia virus 1 (FgBV1)	PQ000867	2525	Fusarium asiaticum ourmiavirus 1	75.9	99	*P_Lenarviricota;C_Miaviricetes;O_Ourlivirales;F_Botourmiaviridae*
Fusarium mitovirus 1 (FMito1)	PQ000868	2531	Erysiphe necator associated mitovirus 6	93	98	*P_Lenarviricota;C_Howeltoviricetes;O_Cryppavirales;F_Mitoviridae*
Fusarium mitovirus 2 (FMito2)	PQ000869	2646	Plasmopara viticola lesion associated mitovirus 13	95	96	*P_Lenarviricota;C_Howeltoviricetes;O_Cryppavirales;F_Mitoviridae*
Fusarium equiseti mitovirus 1 (FeMV1)	PQ000870	2394	Plasmopara viticola lesion associated mitovirus 7	96.9	94	*P_Lenarviricota;C_Howeltoviricetes;O_Cryppavirales;F_Mitoviridae*
Fusarium culmorum flexivirus 1 (FcFv1)	PQ000871	1133+1099+2389+1260+3154	Fusarium boothii large flexivirus 1 Ep-BL13	46–84	87–99	*P_Kitrinoviricota;C_Alsuviricetes;O_Tymovirales*
Fusarium culmorum ormycovirus 1 (FcOMV1)	PQ000872	2529	Trichoderma tomentosum ormycovirus 1	54.66	98	*"Ormycovirus"*
	PQ000873	1826				
Fusarium culmorum yue-like virus 1 (FcYV1)	PQ000874	3205	Plasmopara viticola lesion associated Yue-like virus 3	23.8	67	*P_Negarnaviricota;C_Yunchangviricetes;O_Goujianvirales*
Fusarium culmorum phenuivirus 1 (FcPV1)	PQ000875	6682	Grapevine associated cogu-like virus 1	90	98	*P_Negarnaviricota;C_Ellioviricetes;O_Bunyavirales;F_Phenuiviridae;G_"Bocivirus"*
	PQ000876	1160				
	PQ000877	1198				
Fusarium mononegavirus 1 (FMV1)	PQ000878	8903	Plasmopara viticola lesion associated mononega virus 1	75	98	*P_Negarnaviricota;C_Monjiviricetes;O_Mononegavirales;F_Mymonaviridae;G_Sclerotimonavirus;S_Sclerotimonavirus betaplasmoparae*
Fusarium mononegavirus 2 (FMV2)	PQ000879	7585	Plasmopara viticola lesion associated mononega virus 2	75	98	*P_Negarnaviricota;C_Monjiviricetes;O_Mononegavirales;F_Mymonaviridae;G_Sclerotimonavirus;S_Sclerotimonavirus betaplasmoparae*
Fusarium culmorum mycoophiovirus (FcMOV1)	PQ000880	8616	Trichoderma hamatum mycoophiovirus 1	53.15	94	*P_Negarnaviricota;C_Milneviricetes;O_Serpentovirales;F_"Mycoaspiviridae"*
	PQ000881	2184				
Fusarium culmorum partitivirus 1 (FcPartiti1)	PQ000882	1783	Fusarium mangiferae partitivirus 2	94	100	*P_Pisuviricota;C_Duplopiviricetes;O_Durnavirales;F_Partitiviridae*
	PQ000883	1558				
	PQ000884	1221				

### 3.1 Positive-sense single-stranded RNA mycoviruses

#### 3.1.1 Ambiguiviridae

Among 16 infected *F. culmorum* isolates, six isolates carried a monosegmented virus which we named Fusarium culmorum ambiguivirus 1 (FcAV1). The virus genome was predicted to have two ORFs coding for 556 aa-long hypothetical protein and 552 aa-long RdRp. The encoded RdRp showed similarity to several unclassified (+)ssRNA fungal viruses that are related to a plant-infecting *Tombusviridae* family*.* Although we could not attribute a function to ORF1, DeepTMHMM software (Hallgren et al., 2022) predicted eight transmembrane helices in its sequence (Supplementary Fig. S2 A). They have been suggested to help viral replication by localizing replication proteins to membranes, where replication has been shown to take place in *Tombusviridae* (Gunawardene et al., 2017). The transmembrane domains were found in other related viruses too, suggesting their conserved function (Ai et al., 2016; Cañizares et al., 2018; Khan et al*.,* 2022).

Candidate members of Ambiguiviridae family, as proposed by Gilbert et al. (2019), were described to contain amber stop codon (UAG) at the end of the first ORF. This results in a readthrough and subsequent fusion with the following RdRp protein. In contrast, FcAV1 does not use an amber codon; instead, it has an opal stop codon (UGA). Nonetheless, phylogenetic analysis based on aa sequence of the RdRp, positioned FcAV1 within the proposed members of Ambiguiviridae family (Fig. 3). Furthermore, like other candidate members, FcAV1 contains the GDN triad in Motif C of the RdRp (Supplementary Fig. S2B), instead of canonical GDD motif found in *Tombusviridae*. Lastly, Ambiguiviridae, similar to other (+)ssRNA viruses, appear to employ secondary RNA structures, which are thought to have regulatory roles in RNA processing, transcription, translation, and RNA stability (Lim and Brown, 2018). FcAV1 genome was found to include long UTR regions (412 nt 5′ UTR and 845 nt 3′ UTR), which are expected to form stable secondary RNA structures (Supplementary Fig. S2 C and D).

#### 3.1.2 Mycotymoviridae

Among eight mycovirus-infected *F. graminearum* isolates, five contained a 7886 nt-long contig. Sequence analysis of this contig revealed four putative ORFs. The first and largest ORF encodes a polyprotein that exhibits sequence similarity to several viruses belonging to *Tymoviridae* family, and particularly Fusarium graminearum mycotymovirus 1 (FgMTV1, isolate SX64) (Li et al., 2016). Based on high nucleotide identity (94.5 %, with 124 different amino acids) and similar genome structure, we consider this viral contig as a variant of FgMTV1, and thus named it FgMTV2. The study conducted by Li et al. (2016) suggested that it belongs to a newly proposed genus Mycotymovirus of the *Tymoviridae* family. As is common in *Tymoviridae*, FgMTV2 also has a high cytidine content of 35.7 %, but similarly to FgMTV1 and members of genus *Maculavirus*, FgMTV2 lacks the subgenomic RNA promoters known as “tymobox” and “marafibox”.

The polyprotein of FgMTV2 has been found to contain four conserved domains: viral methyltransferase, endopeptidase, RNA helicase and RdRp, which are all typically conserved in *Tymoviridae* (Martelli et al., 2002). ORFs 2–4 in FgMTV1 have been previously described as having an unknown function (Li et al., 2016). We were unable to determine the functional roles for ORFs 2 and 3, and while BLASTp could not find homologues for ORF 4 either, Phyre2 (90.1 % confidence), Motif (*e* value 0.0016), HHpred (*e* value of 1.6e-5, probability 98 %), and FoldSeek (*e* value of 7.08 × 10^−10^) all showed high-confidence similarity to tymoviral coat protein (CP). This suggests that FgMTV 1 and 2 virions might not be coatless as previously believed.

The phylogenetic tree based on the CP (Fig. 4) clearly illustrates the relationship between FgMTV isolates and other members of *Tymoviridae* family. The tree also supports the Mycotymovirus genus proposed in the earlier work (Li et al., 2016) which was based on the phylogenetic study of the RdRp gene. Both FgMTV isolates were placed in a separate clade from already established genera, together with a few not-yet characterized viruses from *Tymovirales* order.

Notably, all five *F. graminearum* isolates that had FgMTV2 also consistently had two additional viruses, Fusarium mitovirus 2 and Fusarium graminearum botourmia virus 1.

#### 3.1.3 Narnaviridae

We found two *Narnaviridae*-like contigs in five *F. culmorum* isolates. These contigs, correspond to two segments of the same virus, which we have named Fusarium culmorum narnavirus 1 (FcNV1). The first segment (RNA 1) showed 50 % aa identity to RdRps of three orfanplasmoviruses (Chiapello et al., 2020a), with a subsequently lower identity to a few other narna-like RdRps. Phylogenetic analysis based on its aa sequence placed FcNV1 close to several recently reported narna-like viruses (Chiapello et al., 2020a; Jia et al., 2021; Sutela et al., 2021), many of which are multi segmented. However, FcNV1 was shown to be more distantly related to the already classified narnaviruses (Supplementary Fig. S3 A). On the other hand, RNA 2 aa sequence of FcNV1 showed similarity to hypothetical proteins from the second segment of the same viruses. The function of the RNA 2 product of some of these narna-like viruses is pointing towards putative coat protein (Jia et al., 2021). Our Phyre2 search, on the other hand, showed FcNV1 HP from RNA 2 having low-confidence similarity to ribonucleoprotein (Supplementary Fig. S3 B).

#### 3.1.4 Botourmiaviridae

We identified two botourmiaviruses, which we named Fusarium culmorum botourmiavirus 1 (FcBV1) and Fusarium graminearum botourmiavirus 1 (FgBV1), as each of these viruses were found exclusively in either *F. culmorum* or *F. graminearum* isolates, respectively. Both viruses carried one ORF that encoded for RdRp. FcBV1 and FgBV1, along with their top three closest BLASTp matches, formed a cluster with other botourmiaviruses (Supplementary Fig. S4), although they belonged to different clades. FcBV1 showed highest aa identity (73.7 %) to Fusarium subglutinans ourmia-like virus (FsOV1; unpublished), while FgBV1 is most similar (77 %) to Fusarium asiaticum ourmiavirus 1 (FaOV1) (Huang et al., 2023). Since FcBV1 and FgBV1 share >70 % of aa identity with their closest matches, they are classified under the same (Ayllón et al., 2020), not yet established genera.

It should be noted that the FcBV1 original assembly appeared to be chimeric. Therefore, we aligned the sequence to its closest match (OQ565623.1) and trimmed the 5’ end of the contig to a matching length.

#### 3.1.5 Mitoviridae

Three mycoviruses showing strong sequence similarity to the members of the *Mitoviridae* family were found. They share <40 % aa identity among each other but exhibit over 90 % aa identity to other fungal mitoviruses. Therefore, these viruses are classified as belonging to the same species as previously discovered, but not yet classified, viruses. One was found exclusively in *F. equiseti*, thus named Fusarium equiseti mitovirus 1 (FeMito1). The second mitovirus, which we named Fusarium mitovirus 1 (FMito1) was found in three *F. culmorum* isolates, but its partial sequence was also found in *F. graminearum*. Finally, the third mycovirus was found in six *F. graminearum* and one *F. culmorum* isolate and was named Fusarium mitovirus 2 (FMito2). Typical for mitoviruses, all three of them contained one large ORF coding for putative RdRp and had UTR regions at both 5’ and 3’ ends, suggesting that their viral genome sequences are nearly complete.

#### 3.1.6 Flexivirus

**Fig. 2:**
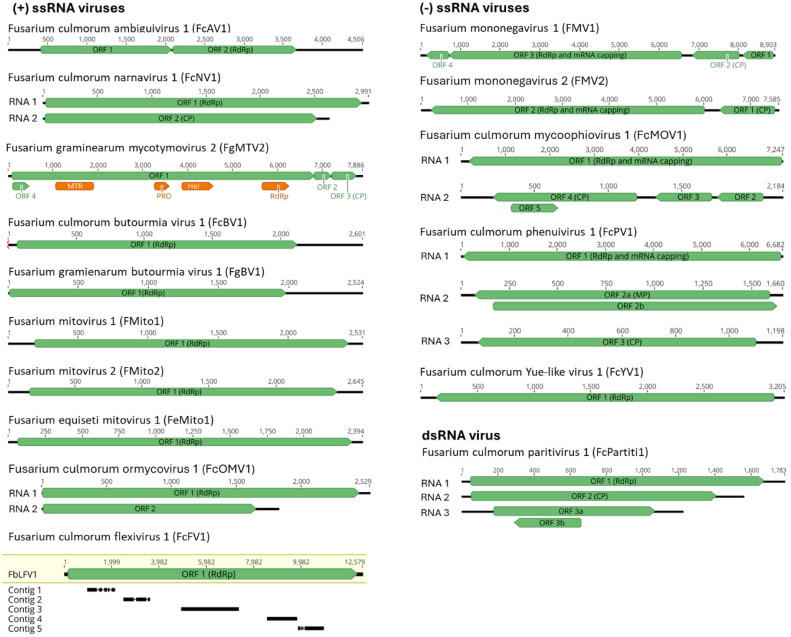
Diagrams illustrating genome structures and putative gene functions of mycoviruses discovered in this study. Black lines represent the sequences of the respective contigs, and green boxes represent open reading frames (ORFs). For FcFV, a Geneious assembly of nucleotide sequences from five contigs and their closest relative, Fusarium boothii large flexivirus 1 (FbLFV1, highlighted in yellow), is depicted, with thin black lines depicting disagreements in alignments. RdRp – RNA-dependent RNA polymerase; MTR – methyltransferase; PRO – protease; HEL – helicase; CP – coat protein; MP – movement protein.

We also identified five contigs, ranging between 1024 and 2389 nt in length, showing similarity to Fusarium boothii large flexivirus 1 (FbLFV1) (Mizutani et al., 2018). Those contigs appear to be partial sequences of a single mycovirus, which we have named Fusarium culmorum flexivirus 1 (FcFV1). The contigs shared between 57 and 74% nt identity with FbLFV1. FbLFV1 has a length of 12.5 kb, and based on the close similarity, we estimate that approximately half of the FcFV1 genome has been sequenced (6.9 out of 12.5 kb, Fig. 2), preventing us from performing further analysis. FbLFV1 itself is unclassified but likely belongs to *Tymovirales* order, sharing 35 % aa similarity to a virus from *Gammaflexiviridae* family.

**Fig. 3:**
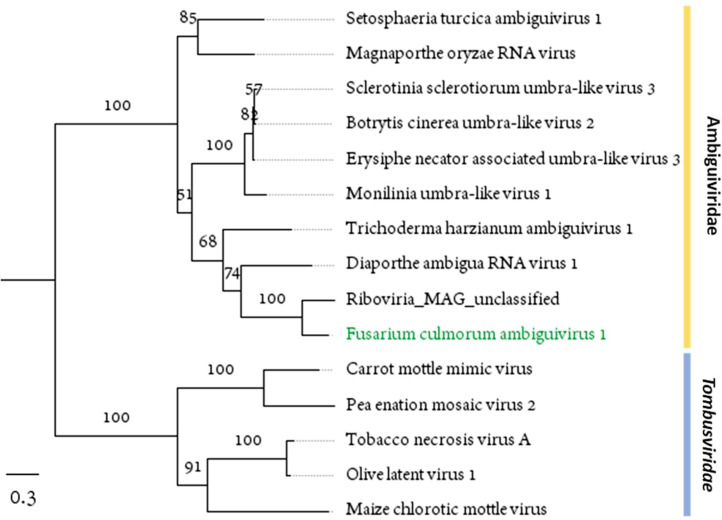
Maximum-likelihood tree depicting the relationships of the predicted aa sequence of RdRp of the FcAV1 and related viruses. Model of substitution: LG+*F* + *I* + G4. Branch lengths are scaled to the expected underlying number of amino acid substitutions per site. Numbers indicate the percentage of bootstrap replicates that support each branch node. See Supplementary Table S3 for viral protein accession numbers.

**Fig. 4:**
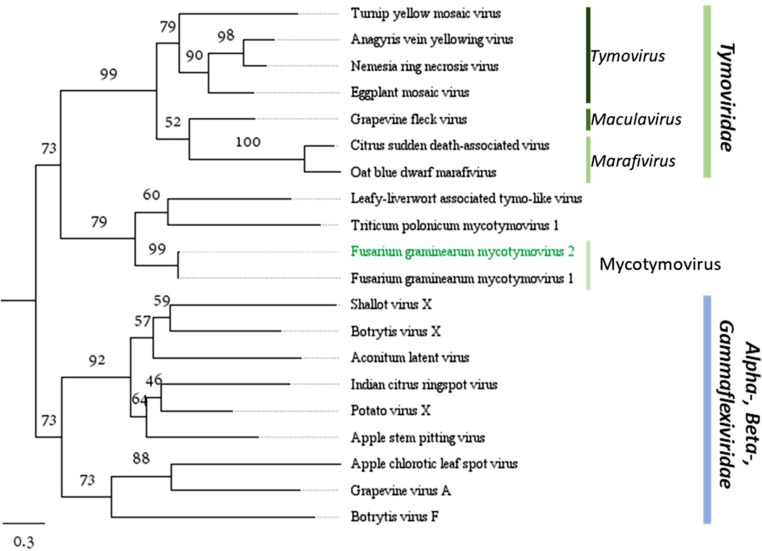
Maximum-likelihood tree depicting the relationships of the predicted aa CP sequence of the FgMTV2, related unclassified viruses, and representatives of all families from *Tymovirales* order and all *Tymoviridae* genera. Model of substitution: VT+G4. Branch lengths are scaled to the expected underlying number of amino acid substitutions per site. Numbers indicate the percentage of bootstrap replicates that support each branch node. See Supplementary Table S3 for viral protein accession numbers.

All five contigs were found in a single *F. culmorum* isolate, but their partial sequences were also detected in one *F. graminearum* isolate (with each contig coverage between 0.3–0.6x).

#### 3.1.7 Ormycovirus

Four *F. culmorum* isolates were found to harbor a virus related to ormycoviruses, a recently described divergent group of bisegmented ssRNA viruses (Forgia et al., 2022). No true virions could be associated with ormycoviruses, preventing their exact classification into the Baltimore system, but a higher positive-sense RNA strand accumulation has been observed, and therefore they are tentatively suggested to belong to (+)ssRNA viruses (Forgia et al., 2022). We named this virus Fusarium culmorum ormycovirus 1 (FcOMV1). The first segment of FcOMV1 is 2.5 kb-long and hosts a single 813 aa-long ORF coding for a putative RdRp. The second segment codes for a hypothetical protein not showing significant similarity to any known proteins. Both RNA 1 and RNA 2 were predicted to form stable secondary RNA structures at 3’ UTR (Supplementary Fig. S5, A and B, respectively).

**Fig. 5:**
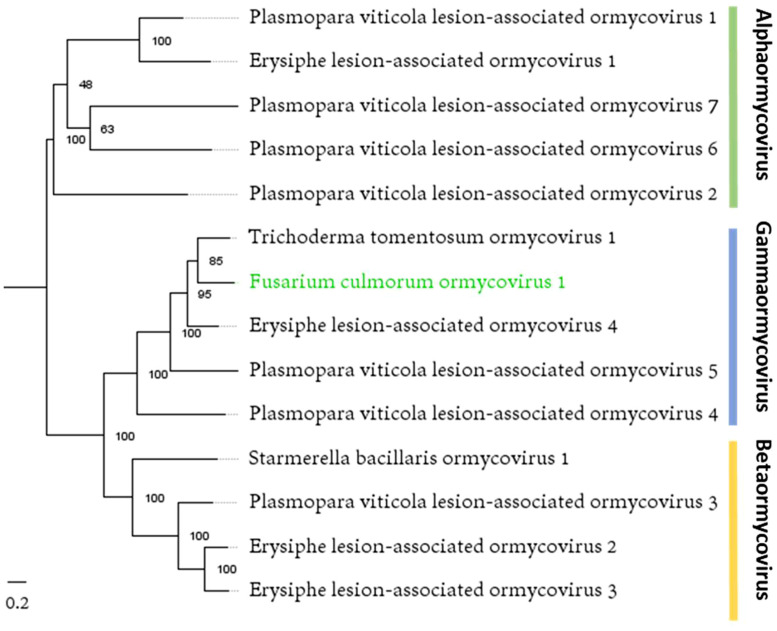
Maximum-likelihood tree depicting the relationships of the predicted aa sequence of RdRp of the FcOV1 and other ormycoviruses. Model of substitution: VT+*F* + *I* + G4. Branch lengths are scaled to the expected underlying number of amino acid substitutions per site. See Supplementary Table S3 for viral protein accession numbers.

Phylogenetic analysis placed FcOV1 in gammaormycovirus clade (Fig. 5), and the position was confirmed by finding GDQ instead of GDD catalytic triad in motif C (Supplementary Fig. S5 C), a characteristic unique to gammormycoviruses (Forgia et al., 2022).

### 3.2 Negative-sense single-stranded RNA mycoviruses

#### 3.2.1 *Yueviridae*-like

**Fig. 6:**
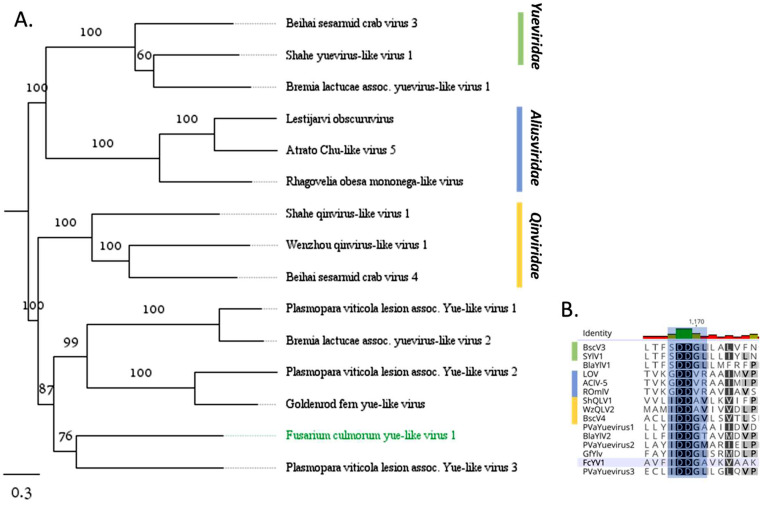
A. ML tree depicting the relationships of the predicted aa sequence of ORF encoding RdRp in FcYV1 and related viruses. The viruses were selected from BLAST hits, representatives of *Yueviridae, Aliusviridae, Quinviridae*, as well as similar Yue-like viruses from oomycetes. The best-fit model was found to be Blosum62+*F* + R3. Sequence information of all selected viruses was supplied in Supplementary Table S3. The numbers next to each branch represent the bootstrap support based on 1000 replicates. All branch lengths are drawn to a scale of amino acid substitutions per site. B. Motif C of RdRp from FcYV1 and related viruses. The catalytic triad of motif C is shaded in blue.

Fusarium culmorum yue-like virus 1 (FcYV1) was found in four *F. culmorum* isolates, each of which was also infected with 4–6 other viruses. We found one 3.5 kb-long segment of FcYV1 genome, consisting of one ORF, coding for a putative RdRp. BLASTx showed significant similarity to a virus belonging to the *Yueviridae* family, two viruses from the *Aliusviridae*, as well as several yue-like unclassified viruses (all up to 25 % sequence identity). Phylogenetic tree (Fig. 6A) showed that FcYV1, together with several recently described yue-like oomycete viruses (Chiapello et al., 2020a; Forgia et al., 2024), constitute a distinct clade from other previously classified viruses related to viruses belonging to *Qinviridae* family*.* All of them possess a conserved IDD tripled in the Motif C of catalytic core of the RdRp, as opposed to the SDD found in the members of the *Yueviridae* and the GDD in the *Aliusviridae* family (Fig. 6B).

#### 3.2.2 Phenuiviridae

A tripartite virus from *Phenuiviridae* family was detected in five *F. culmorum* isolates; although, the second segment of the virus was not detected in one of the isolates. We named the virus Fusarium culmorum phenuivirus 1 (FcPV1). The first segment contains an ORF with a *Bunyavirales* RdRp domain. It showed 90 % aa identity to the RdRp of grapevine associated cogu-like virus 1 (GaCLV1) (Chiapello et al., 2020b). Its RNA 2 harbored two overlapping ORFs (ORF 2a and 2b) in ambisense orientation. ORF 2a putatively codes for a movement protein (MP), as evidenced by BLASTp showing 30 % similarity to MP of citrus virus A, as well as HHpred finding a core domain from 30 K type MP (*e*-value 0.00093, pfam PF17644). ORF 2b, on the other hand, did not return any significant hits in any of the tools. Finally, a *Tenuivirus*/*Phlebovirus* nucleocapsid protein domain was found in RNA 3 (PF05733 *e*-value 4.4e-43 in HHpred, 2.01e-06 in CDD).

Ruiz-Padilla et al. (2021) proposed a new genus, *Bocivirus,* to encompass GaCLV1 and their newly discovered mycovirus, Botrytis cinerea bocivirus 1 (BcBV1), which was recently accepted by ICTV. Our phylogenetic analysis, based on the aa sequence of RdRp gene (Supplementary Fig. S6 A), demonstrates a distinct cluster for FcPV1, GaCLV1 and BcBV1, diverging from previously classified plant coguviruses, in line with Ruiz-Padilla et al. (2021). A CP-based phylogenetic analysis shows a tree similar to the RdRp-based one, where bociviruses and coguviruses form a separate clade but still share a more recent common ancestor than laulaviruses (Supplementary Fig. S6 B). A phylogenetic tree based on MP, on the other hand, shows the bocivirus clade clustering closer to laulaviruses, rather than coguviruses (Supplementary Fig. S6 C). Furthermore, FcPV1 and GaCLV1 MPs form a distinct clade from the other bociviruses. Taken together, the phylogenetic analyses support the placement of FcPV1 within the *Bocivirus* genus. Moreover, since the demarcation criteria for species within *Bocivirus* were established as <95 % identity in the aa sequence of the RdRp, FcPV1 qualifies as a member of distinct species.

#### 3.2.3 Mymonaviridae

A pair of related contigs sharing 92.8 % nucleotide identity was found. The longer one had four ORFs, whereas the shorter one appeared to be truncated, carrying only two ORFs. We named the viruses Fusarium mononegavirus 1 and 2 (FMV1 and FMV2). The longest ORF of both contigs contained ∼1940 aa-long RdRp with *Mononegavirales* RdRp l-protein (pfam00946) catalytic domain and PF14318, mRNA-capping region V (pfam14318). In addition to RdRp, FMV1 isolate also harbored nucleoprotein and two ORFs coding for hypothetical proteins, whereas FMV2 isolate only encoded nucleoprotein. HHpred detected coiled coil segments in HP from ORF 4 of FMV1, and a similarity to reovirus outer capsid protein sigma-1 (PDB structure 6GAP) indicating that it may serve a structural role.

Based on the taxonomic classification of the closest relative, Plasmopara viticola lesion associated mononegavirus 2 (PvLamononegaV2) (Chiapello et al., 2020a), which shares 75.5 and 75.1 % aa identity with FMV1 and FMV2, respectively, these viruses were determined to belong to the *Sclerotimonavirus* genus and the *Sclerotimonavirus betaplasmoparae* species within the *Mymonaviridae* family. This classification follows species demarcation criteria of >30 % similarity in nucleoprotein amino acid sequences (FMV1/2 and PvLamononegaV2 share > 77 %) and a coding-complete genome nucleotide sequence similarity of over 30 % (> 67 %) (Jiāng et al., 2022). FMV1 was present in four isolates of *F. culmorum* and one isolate of *F. graminearum*, while FMV2 was found in one *F. culmorum* isolate.

#### 3.2.4 Mycoaspiviridae

Another virus, hosting the *Mononegavirales* RdRp domain and mRNA capping region V in its ORF 1, was found in nine *F. culmorum* isolates and partially in one *F. graminearum* isolate. This virus was designated as Fusarium culmorum mycoophiovirus 1 (FcMOV1). The top closest matches in BLASTp were mycoviruses related to *Aspiviridae* family (formerly *Ophioviridae),* with the highest being Suillus luteus mycoophiovirus 1 (Liu et al., 2023) (53 % aa identity)*.* Chiapello et al. (2020a) proposed to call this clade of *Aspiviridae*-related fungi-infecting viruses Mycoaspiviridae. Our phylogenetic analyses confirmed FcMOV1 placement in the proposed Mycoaspiviridae family (Supplementary Fig. S7 A).

Furthermore, we found a contig that could be putatively assigned as RNA 2 of FcMOV1, based on its similarity (57 % aa identity over 41 % query cover) to RNA 2 of Colletotrichum associated negative-stranded RNA virus 2 (Hamim et al., 2022), which is another member of the proposed Mycoaspiviridae family. Additionally, the two FcMOV1 contigs were found to be present together in the same fungal isolates, apart from one isolate which showed only 0.1x coverage of RNA 1 but 0.6x coverage of RNA 2 (Supplementary Table S1). Four ORFs longer than 300 nt could be detected in the contig of RNA 2. While their functional assignment was challenging, HHpred showed strong homology between ORF 4 and nucleocapsid proteins of two *Aspiviridae* viruses, namely Mirafori lettuce big-vein virus and citrus psorosis virus (Supplementary Fig. S7 B).

### 3.3 Double-stranded RNA mycovirus

#### 3.3.1 Partitiviridae

Finally, seven *F. culmorum* isolates were found to carry a tri-segmented virus belonging to the *Partitiviridae* family. We named the virus Fusarium culmorum partitivirus 1 (FcPartiti1). Due to infecting fungi and characteristic lengths of RNA 1 and RNA 2, the virus likely belongs to *Gammapartitivirus* genus. RNA 1 hosted an ORF coding for RdRp, which shared 94 % aa identity with Fusarium mangiferae partitivirus 2 (Khan et al., 2021). The RNA 2 segment hosted a CP gene with 77 % aa identity to Fusarium oxysporum partitivirus 1. Finally, RNA 3, which was absent from two isolates carrying FcPartiti1, carried two putative ORFs that were overlapping and oriented in ambisense. The longer one (ORF 3a) shared 42 % similarity with HP from Plasmopara viticola lesion associated partitivirus. However, the function of either of the ORFs in RNA 3 could not be determined.

The criteria for distinguishing species within the genus *Gammapartitivirus* are <90 % aa sequence identity in the RdRp and <80 % in the CP. Based on the observed lower aa sequence identity in the CP than the required threshold, it is likely that a new species needs to be established.

## 4 Discussion

*F. culmorum* is a plant pathogen with worldwide prevalence, particularly known for its ability to cause foot and root rot and FHB (Scherm et al., 2013). Alongside *F. graminearum* and *F. avenaceum, F. culmorum* ranks as the most common species causing FHB in Denmark (Nielsen et al., 2011). However, in contrast to the other two species, mycoviruses in *F. culmorum* remain largely unexplored, despite intensified efforts in recent years to unveil mycoviruses in various fungal species. In this work, we focused on the mycovirome of primarily *F. graminearum* and *F. culmorum* obtained directly from wheat fields. Our aim was to unravel distribution and diversity of fungal viruses, which could eventually lead to the development of a potential biological control agent. We identified 16 different mycoviruses, six of which were multisegmented, and they belonged to four out of six currently recognized phyla in the kingdom *Orthornavirae: Kitrinoviricota, Lenarviricota, Negarnaviricota* and *Pisuviricota.*

### 4.1 Variations in mycovirus distribution

While the number of sequenced fungal isolates was similar, mycoviruses were more common in *F. culmorum* (16/29 carried viruses) than in *F. graminearum* (8/24). Notably, all isolates containing many mycoviruses (five or more) were associated with *F. culmorum*. Possible reasons for the discrepancy could include low diversity in *F. culmorum* vegetative compatibility groups (VCG) in the sampled experimental field, facilitating easier sharing of mycoviruses between different strains. Multiple studies have demonstrated a high degree of VCG diversity in *F. graminearum* (McCallum et al., 2001; Ramirez et al., 2006), however, to our knowledge, no such studies exist about *F. culmorum*. The variation in virus diversity may also be related to the differences in the means of reproduction between the two species*. F. graminearum* is known to undergo both sexual and asexual reproduction, whereas in *F. culmorum* only asexual reproduction is known (Leslie and Summerell, 2006b). The production of ascospores during sexual reproduction has been shown to result in a loss of viruses in other fungi (Carbone et al., 2004), and may serve as a method to eliminate viruses in *F. graminearum*, too. Finally, another possible explanation is that one or several mycoviruses present in *F. culmorum* may down-regulate the incompatibility reactions and allow mycoviruses to be transferred even between vegetatively incompatible strains, which has been demonstrated for Sclerotinia sclerotiorum mycoreovirus 4 (Wu et al., 2017).

Unsurprisingly, the occurrence of mycovirus infection was less prevalent in fungi isolated from wheat plots with artificially inoculated *Fusarium* spp. compared to fungi isolated from wheat plots where FHB infection occurred naturally (40 % versus 63 %). Furthermore, the artificially inoculated plots also experienced natural infection from the surroundings, which could explain why not all isolates from artificially FHB-infected wheat carried the same viruses. Nevertheless, this suggests that mycovirus transmission is dynamic and has the potential to affect fungal interactions within the same growing season. To elucidate this further, fungal isolates from the culture collection used in the artificial inoculation could be screened for the presence of mycoviruses in a future study.

Given that single spore isolation is a strategy for curing viruses (Khan et al., 2023), we stipulated that obtaining *Fusarium* spp. isolates via single spore isolation could unintentionally cure some isolates of mycoviruses. To avoid this, some of them were obtained by subculturing hyphae instead. The results suggest that this had a negligible effect, with 45 % of isolates obtained via single spore isolation carrying mycoviruses, compared to 50 % of isolates obtained with hyphae subculturing method. Nonetheless, as most of the isolates were obtained via single spore isolation (40 out of 54), the dataset is too small to draw strong conclusions.

The biggest influence on mycovirus presence and diversity was the wheat plot the fungus was isolated from (the combination of wheat cultivar, year, and infection source). Perhaps a larger fungal variability (less clonal isolates) within wheat plots and a higher mycovirus diversity could have been obtained if several different media were used. We used PDA for obtaining the majority of the isolates, however, media such as SNA that induces sporulation in a broader number of isolates could possibly have been a better choice (Moura et al., 2020).

Another interesting finding is that some of the mycoviruses appear to be present in both *F. graminearum* and *F. culmorum*. Specifically, it was FMito1, FMito2, FcFV1, FMV1 and FcMOV1. The presence of shared mycoviruses suggests that several recent cross-species mycovirus transfers occurred. Furthermore, in four cases mycovirus contig coverage in *F. graminearum* was lower than in *F. culmorum*. A possible reason for the lower coverage could be a lower viral titer, and, if true, it could suggest that these mycoviruses can replicate in *F. culmorum* more efficiently than in *F. graminearum*. Regardless, mycovirus’ ability to infect several related taxa could be a valuable feature in a potential biological control agent. A recent study provided compelling evidence that mycovirus transmission between fungal strains is facilitated by plants (Hai et al., 2024). While it is unclear if the same plant effect could apply to interspecies transmission, other studies have observed mycoviruses in several different host species as well (Pagnoni et al., 2023; Vainio et al., 2010).

Previously, dsRNA viruses were considered to be the most common type, but recently the paradigm changed to (+)ssRNA viruses (Ayllón and Vainio, 2023). Our study also found (+)ssRNA viruses to be the most common in *Fusarium* species, followed by (-)ssRNA viruses. This finding is still rather unexpected, given that we utilized a dsRNA enrichment method, which typically underrepresents (-)ssRNA viruses (Weber et al., 2006). Instead, dsRNA genomes were the rarest, with only one dsRNA virus discovered. Additionally, viruses from the phylum *Duplornaviricota*, consisting of dsRNA viruses and therefore expected to be abundant in the samples, were completely absent.

### 4.2 Intriguing mycoviruses reported

In our (+)ssRNA virus group, several interesting mycoviruses were found. Firstly, we identified a new virus from the proposed Ambiguiviridae family not yet recognized by the ICTV. Additionally, we detected a virus with strong similarity to FgMTV1, which previously has been shown to cause mild hypovirulence (Li et al., 2016). FgMTV1 was isolated from China, making it unexpected to observe a 94.5 % nucleotide identity match with FgMTV2, which we isolated in Denmark. We speculate that the widespread distribution of wheat seeds through global commerce could account for this small sequence divergence. Originally, FgMTV1 was reported to lack a CP; however, several tools revealed homology between proteins of unknown function from FgMTV 1 and 2 and coat proteins from other *Tymoviridae* members. This suggests that fungal tymoviruses, proposed to be named mycotymoviruses, may not be as distant from their plant virus counterparts as initially believed.

Furthermore, we found an unusual narnavirus with two genome segments. Conventionally, the family *Narnaviridae* is considered to be among the simplest monosegmented viruses, but recently several related bi-segmented, tetra-segmented, and even negative-strand coding narna-like viruses were discovered (Dinan et al., 2020; Jia et al., 2021; Sutela et al., 2021). Although only two narnaviruses are officially recognized by ICTV, our phylogenetic analysis placed orphanplasmovirus-related viruses, including FcNV1, on a sister clade of already classified narnaviruses. Therefore, it is evident that viruses from the *Narnaviridae*, orphanplasmoviruses, and other related viruses warrant further attention and additional efforts to discover more strains before the taxonomical classification of *Narnaviridae* can be completed.

We also expanded fungal repertoire of (-)ssRNA viruses by discovering mycoviruses from four different classes. We found yue-like mycovirus, FcYV1, which is to our knowledge the first *Yueviridae- and Qinviridae*-like virus found in true fungi. Related viruses so far were associated with oomycetes and invertebrates (Chiapello et al., 2020a; Forgia et al., 2024; Shi et al., 2016). Notably, our phylogenetic analysis showed that FcYV1, along with oomycete mycoviruses Plasmopara viticola lesion associated Yue-like virus (PVaYuevirus) 1, 2, 3, and Bremia lactucae associated yuevirus-like virus 2, are more closely related to qinviruses, rather than yueviruses, as argued in studies by Chiapello et al. (2020a) and Forgia et al. (2024). The link with *Qinviridae* is further supported by the sharing of IDD catalytic triad in motif C of the RdRp. Lastly, it is important to note that FcYV1 genome likely is not complete. Second and third genome segments were discovered in mycoviruses from *Bremia lactucae* and PVaYuevirus1, but are missing in FcYV1 and PVaYuevirus2 and 3. They lack at least the genomic segment coding for nucleoprotein, which is thought to be required for replication in (-)ssRNA viruses (Ruigrok et al., 2011). The absence of these segments suggests they remain to be detected as more mycoviruses are discovered, potentially bridging the gap between viruses that currently share low sequence similarity.

*Phenuiviridae* family consists of diverse viruses from several hosts. Furthermore, the genome structure and composition of these viruses are quite diverse, suggesting high divergence. Their genomes consist of RdRp- and nuclecapsid-coding genes, but some phenuivirids additionally code for glycoproteins, movement proteins, and RNA silencing suppressor proteins (Sasaya et al., 2023). FcPV1 was found to be an obvious member of this family, with a coding capacity for putative RdRp, nucleocapsid, MP, and a protein of unknown function. MP proteins are common in plant viruses but are suspected to be obsolete for infection in fungi (Buivydaitė et al., 2024). Nonetheless, due to close plant-fungi association in nature, a possible cross-kingdom event has been suggested (Ruiz-Padilla et al., 2021), resulting in a plant virus establishing a successful infection in fungi, and therefore the existence of MP in mycoviruses. It is likely that such protein is not functional in mycoviruses. However, it is interesting that it is still present in most of the phenuivirids infecting different fungal hosts, and that it was lost in one out of four isolates carrying FcPV1 in our study. More studies are indeed needed to investigate the potential function of MP in mycoviruses. Another mystery involving the coding potential of these mycoviruses is the overlapping gene in ambisense orientation present in FcPV, GaCLV1, Sanya phenuivirus 1 and Fusarium sibiricum coguvirus 1. These genes code for a hypothetical protein that shows no homology to other known proteins, and even between themselves (between 5 and 36 % aa identity; Supplementary Fig. S6 D). Therefore, further experiments are needed to confirm if the proteins are actually translated and what their function could be.

The only virus with dsRNA genome was FcPartiti1. *Partitiviridae* is another family of ubiquitous viruses that canonically are assumed to have minimal effects on their hosts. While typically composed of two dsRNA segments, some partitivirids are tripartite (Jiang et al., 2019), with one reported to harbor four segments (Zhang et al., 2013) and another eight segments (Kuroki et al., 2023). At least one of the tripartite viruses, Aspergillus flavus partitivirus 1, causes abnormal growth in vitro (Jiang et al., 2019). Our data suggests that the third segment of FcPartiti1, encoding a protein of unknown function, may not be essential for the transmission and replication FcPartiti1, as two isolates lacked it. Nevertheless, further experiments are warranted to elucidate its precise role.

Finally, we found another instance of fungal virus from a recently discovered ormycovirus viral group. Due to low sequence homology to already established viral taxa, ormycoviruses cannot yet reliably be placed on global RNA virus phylogenetic tree (Forgia et al., 2022). They appear to infect fungi (Pagnoni et al., 2023), oomycetes (Forgia et al., 2022), and possibly even plants (Niu et al., 2024).

## 5 Conclusion

Our study provides novel insights into the diversity and prevalence of mycoviruses among *Fusarium* spp. associated with Fusarium head blight in wheat. Our findings reveal that mycovirus infections are common, particularly in *F. culmorum*. Additionally, the identification and sequence characterization of various novel mycoviruses expand our understanding of the viral diversity within these fungal pathogens. This study represents the first investigation into the mycovirome of Danish isolates of FHB-causing fungi. This could potentially lead to the development of biological control strategies against *Fusarium* spp., as well as a deeper understanding of the roles mycoviruses play in *Fusarium* ecology and disease development.

## CRediT authorship contribution statement

**Živilė Buivydaitė:** Writing – review & editing, Writing – original draft, Visualization, Methodology, Investigation, Conceptualization. **Anne Winding:** Writing – review & editing, Supervision, Project administration, Methodology. **Lise Nistrup Jørgensen:** Writing – review & editing, Supervision, Methodology, Investigation. **Athanasios Zervas:** Writing – review & editing, Methodology. **Rumakanta Sapkota:** Writing – review & editing, Supervision, Methodology, Funding acquisition, Conceptualization.

## Declaration of competing interest

The authors declare that they have no known competing financial interests or personal relationships that could have appeared to influence the work reported in this paper.

## Data Availability

Data submitted to public database.

## Appendix Supplementary materials

Image, application 1

Image, application 2

Image, application 3

Image, application 4
