# Supplementary material for: New insights into RNA mycoviruses of fungal pathogens causing Fusarium head blight
Source: Virus Res. 2024 Sep 13;349:199462. doi: 10.1016/j.virusres.2024.199462 (PMC11417338; doi:10.1016/j.virusres.2024.199462)
Supplement: Supplementary file 1 [file mmc1.docx]

**
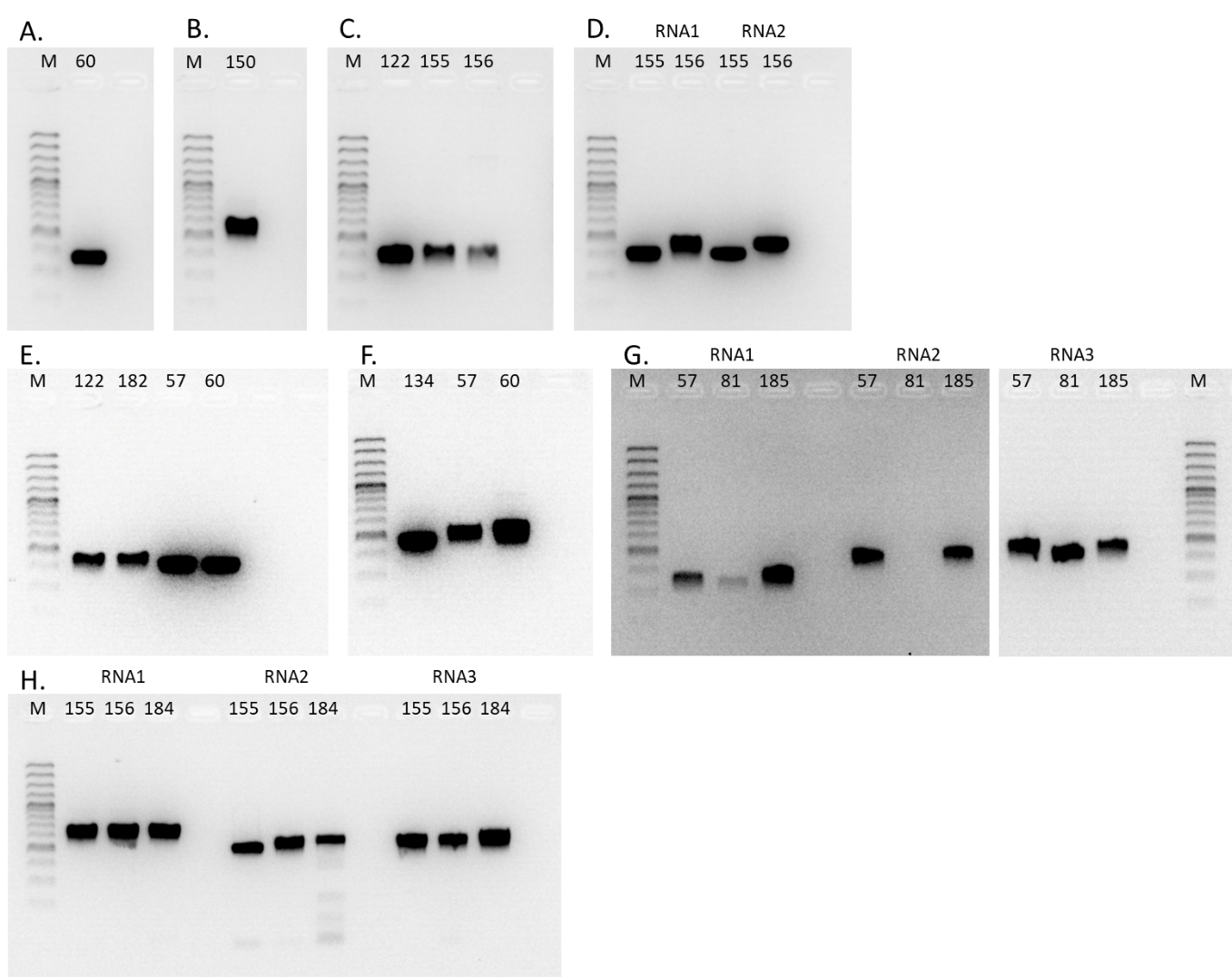
**

*Supplementary Figure S1. Agarose gel electrophoresis results confirming the presence of mycoviruses in selected isolates, as detected by RT-PCR. A.* *Fusarium culmorum mitovirus 1. B. Fusarium culmorum mitovirus 2. C. Fusarium culmorum botourmia virus 1. D. Fusarium culmorum mycoophiovirus 1. E. Fusarium mononegavirus 1. F. Fusarium mononegavirus 2. G. Fusarium culmorum phenuivirus 1. H. Fusarium culmorum partitivirus 1.* *The specific primers used for amplification are detailed in Supplementary Table S2. Lane M contains the DNA size marker (GeneRuler 100 bp Plus, Thermo Scientific) for reference.*

*
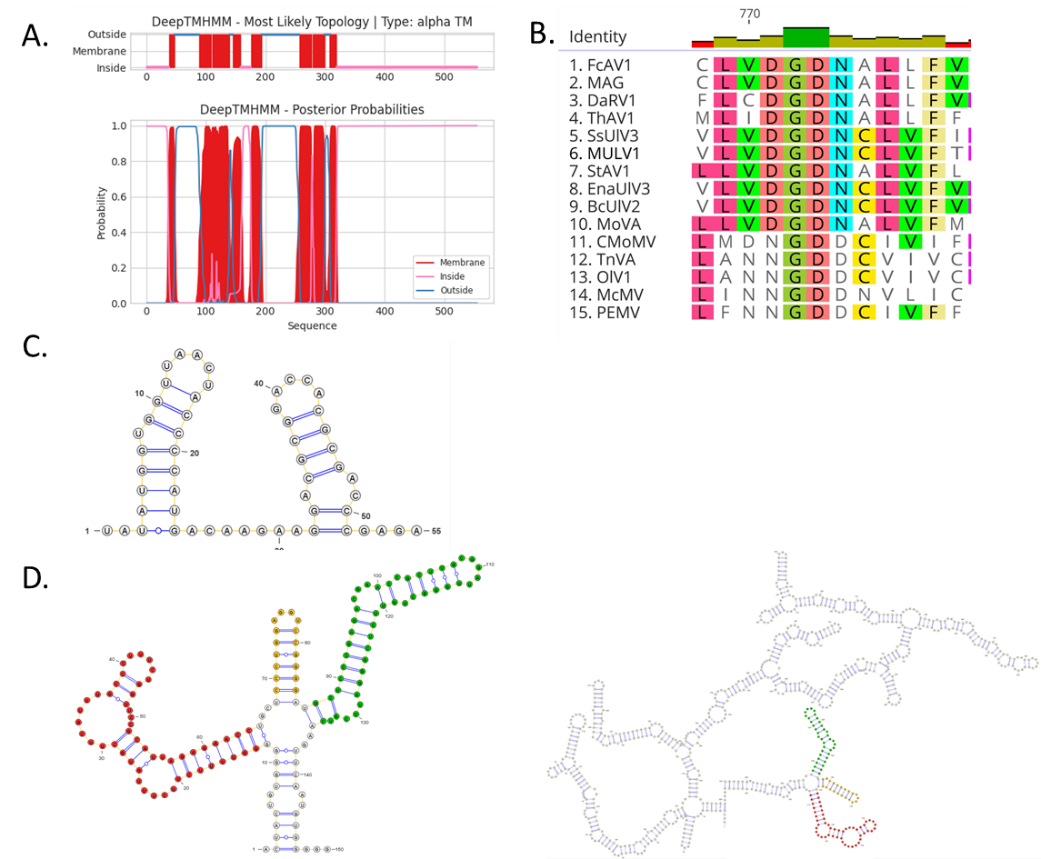
*

*Supplementary Figure S2. A. Six transmembrane helices domains predicted at N-terminus of the ORF1 of FcAV1 using the DeepTMHMM web software. B. Motif C of the RdRp, showing GDN as catalytic triad in Ambiguiviridae, and GDD in Tombusviridae. C. Predicted secondary structure of the terminal 55nt of 5′ UTR of FcAV1 (ΔG=-20.35 kcal/mol. The structure is predicted to form when 3´ terminal 55-220nt are tested. In case of longer terminal sequences, the second stem loop structure is joined with a larger one downstream. D. Predicted secondary structure at the 3′ UTR of FcAV1. Picture on the left illustrates the last 150 nt at the 3′ UTR (ΔG=-59.87 kcal/mol), while the picture on the right illustrates full 845 nt-long 3′ UTR (ΔG=-352.76 kcal/mol). Highlighted RNA structures were consistently observed when progressively larger 3′-terminal portions of the FcAV1 RNA sequences were tested.*


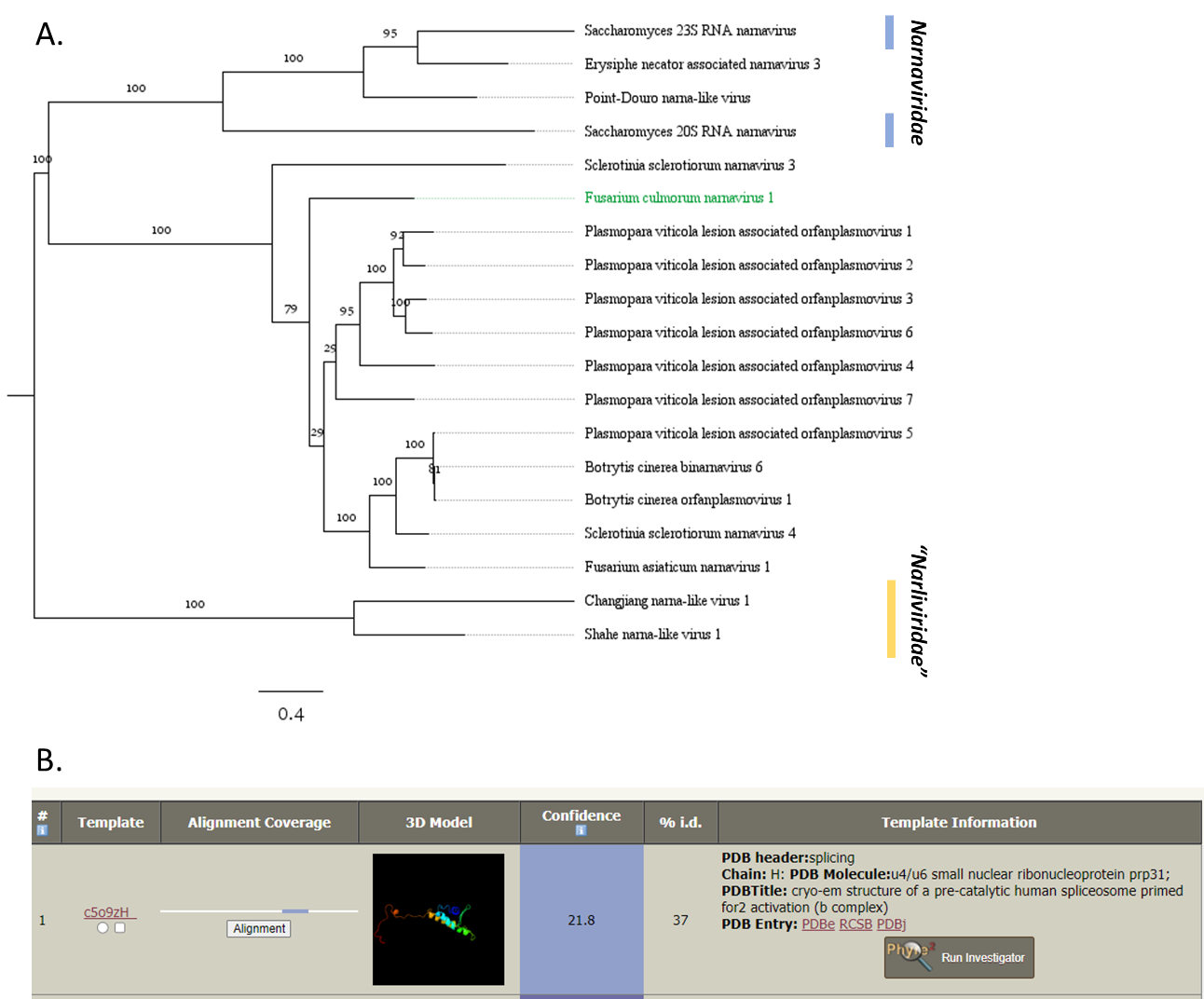


*Supplementary Figure S3. A.* *ML tree showing clustering of FcNV1 (in green) together with other bisegmented narna-like viruses, orfanplasmoviruses, as well as classified and related members of Narnaviridae. The best-fit model was found to be rtREV+F+R4. Sequence information of all selected viruses was supplied in Supplementary Table S3. The numbers next to each branch represent the bootstrap support based on 1000 replicates. All branch lengths are drawn to a scale of amino acid substitutions per site. B. Phyre2 result obtained from RNA 2 aa sequence.*

*
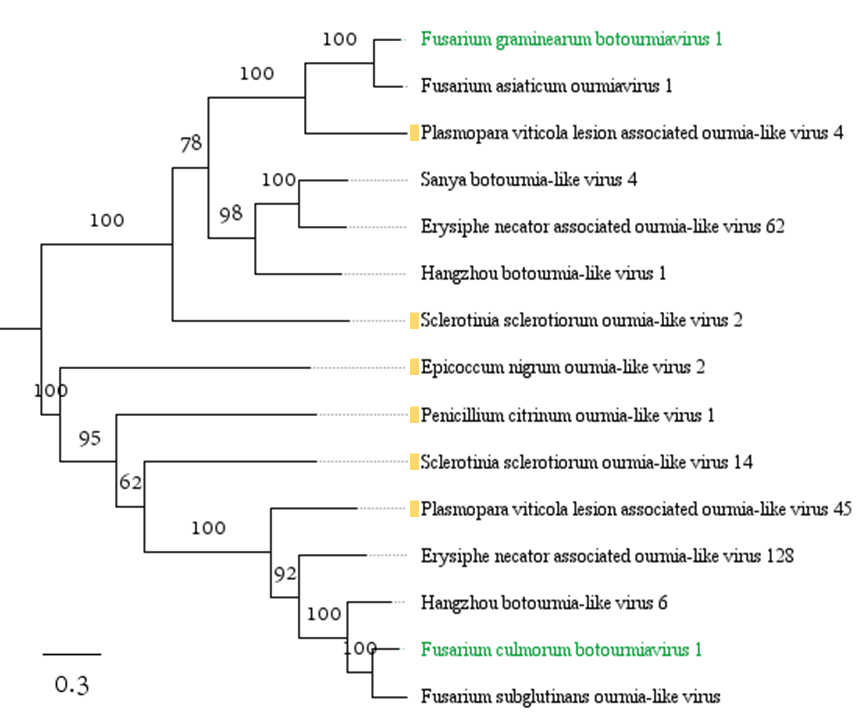
*

Supplementary Figure S4. Maximum-likelihood tree depicting the relationships of the predicted aa sequence of RdRp of the FcBV1, FgBV1 and related viruses. Yellow blocks next to the name abbreviations indicate that the virus is officially classified as Botourmiaviridae by ICTV. Model of substitution: VT+F+R3. Branch lengths are scaled to the expected underlying number of amino acid substitutions per site. Numbers indicate the percentage of bootstrap replicates that support each branch node. See Supplementary Table S3 for viral protein accession numbers.

*
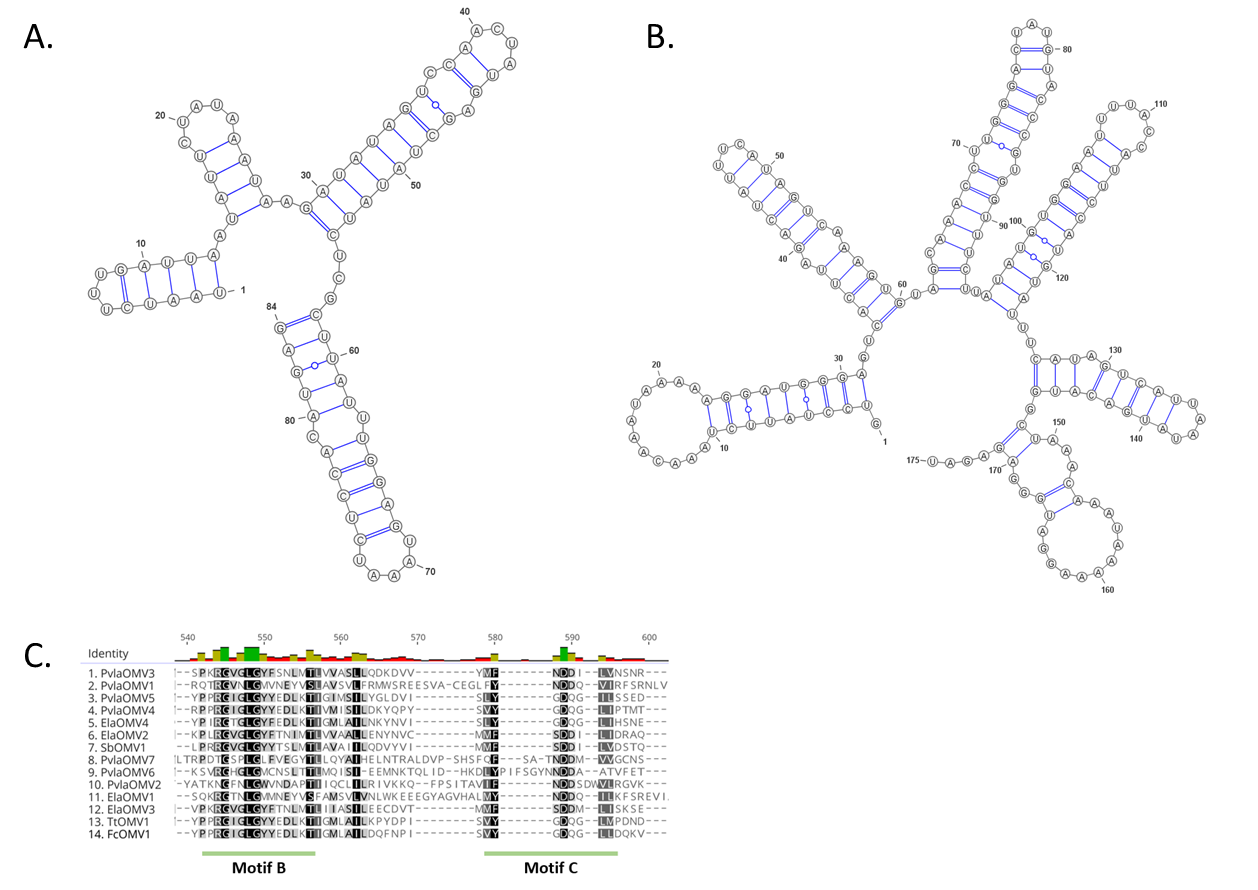
*

*Supplementary Figure S5. A, B. Predicted secondary structure of the* *3′ UTR of FcOV1 RNA 1(A, ΔG=-26.74 kcal/mol) and RNA 2 (B, ΔG = -59.04 kcal/mol). C. Motifs B and C of the RdRp of FcOV1.*


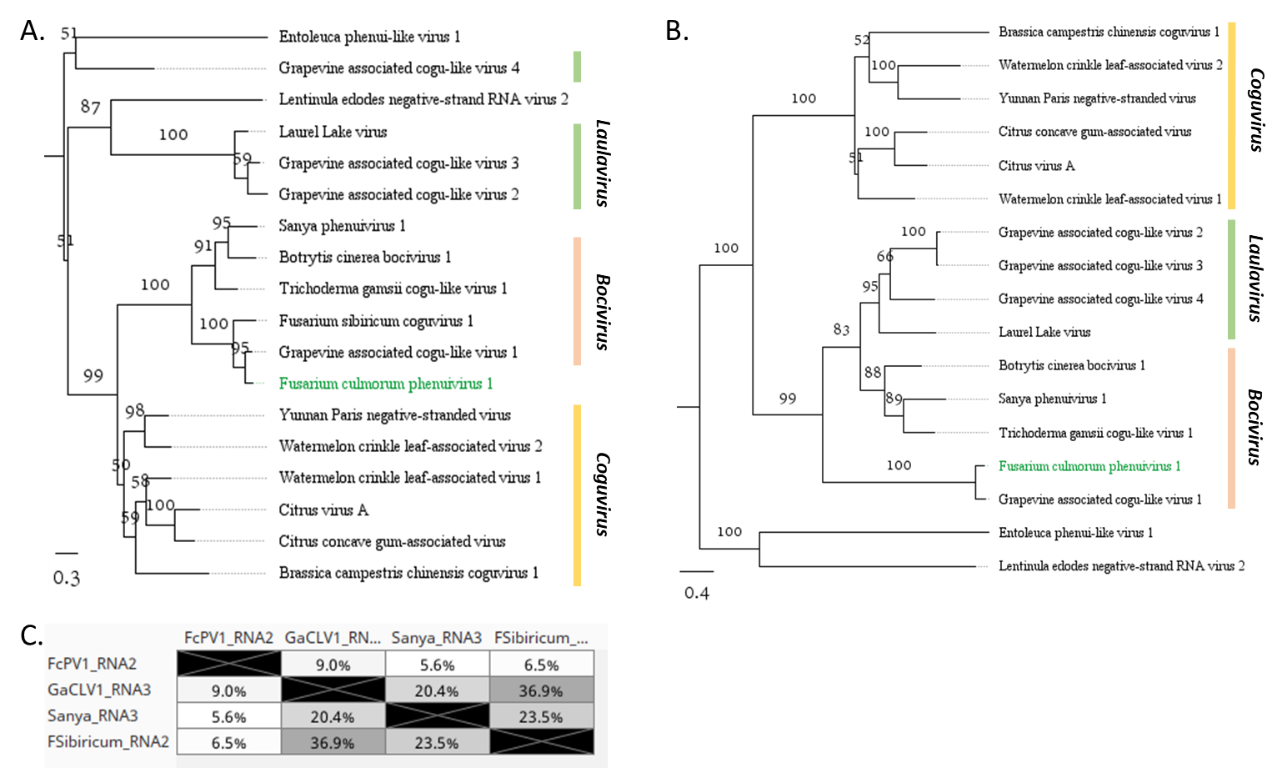


*Supplementary Figure S6. ML trees depicting the relationships of the predicted aa sequence of ORF encoding CP (A) and MP (B) in FcPV1 and related viruses. The best-fit models were found to be LG+F+I+G4 and rtREV+F+R4, respectively. See Supplementary Table S3 for viral protein accession numbers. C. Distance matrix showing % identity between amino acid sequences of the hypothetical protein, based on Muscle alignment.*

**
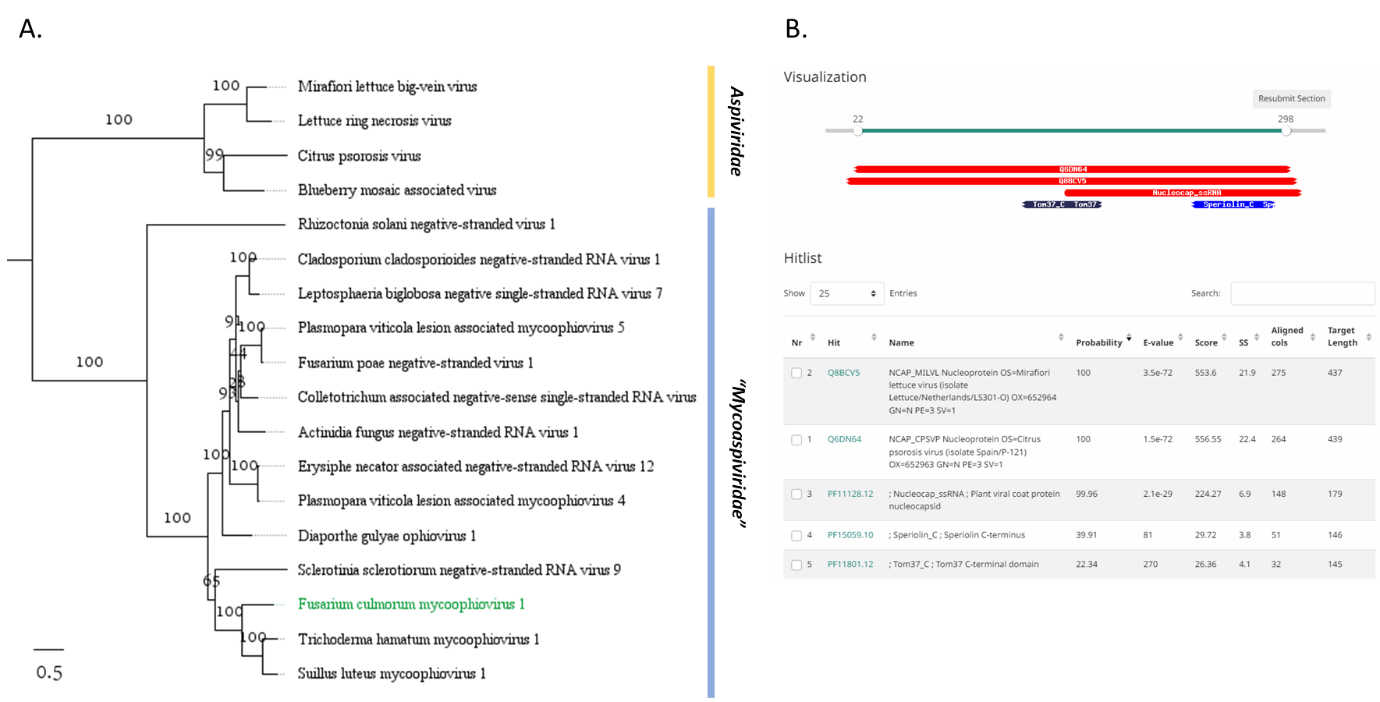
**

*Supplementary Figure S7. A. ML tree depicting the relationships of the predicted aa sequence of ORF encoding RdRp in FcMOV1 and related viruses. The best-fit model was found to be LG+F+R4. See Supplementary Table S3 for viral protein accession numbers. B. Results page from HHpred analysis.*
