# Supplementary material for: New insights into RNA mycoviruses of fungal pathogens causing Fusarium head blight
Source: Virus Res. 2024 Sep 13;349:199462. doi: 10.1016/j.virusres.2024.199462 (PMC11417338; doi:10.1016/j.virusres.2024.199462)
Supplement: Supplementary file 3 [file mmc3.pdf]

Supplementary Table S2. Primers used in RT-PCR.

**Target mycovirus**	**Sequence (5’-3’)**
Fusarium culmorum mycoophiovirus 1 RNA1	ATCGCCCAACTCGGCTTATT
	TCCAGGACCGACCCATTGTA
Fusarium culmorum mycoophiovirus 1 RNA2	AGGTCGTCATGGGTTTGGAC
	TACGATGGCTCACGAACTGG
Fusarium mononegavirus 1	GTCTGGCCTCCTTTCACGAA
	AAACACCGTGCGGATCTCTT
Fusarium mononegavirus 2	CGCCTGTGGGAAGAGAATGT
	GAGACGCGGCATGTTTCATC
Fusarium culmorum botourmia virus 1	AACTCCCGGAAAACCTCGTC
	CCCATTAGTTGGCCTGTCGT
Fusarium culmorum partitivirus 1 RNA1	AGCTTGAACCATTCGTCGGT
	GGAAGAGTGCCCAACGGTTA
Fusarium culmorum partitivirus 1 RNA2	TCCAAGCAGGAAGGGTTTGG
	TCCAACTCCGAAATGCCGTT
Fusarium culmorum partitivirus 1 RNA3	GCCCCTGACAATTGGGATGA
	ATTCCGTCGCCCTTTGCTTA
Fusarium culmorum phenuivirus 1 RNA1	CAGCAGACGCCTCAAAATGG
	CCTGGATCACGCCATGAGTT
Fusarium culmorum phenuivirus 1 RNA2	ATCCAGGTCATCAGTGGGGA
	TTCCTGTGGGTCTCAACAGC
Fusarium culmorum phenuivirus 1 RNA3	AGCTGCAGCAACTCTAGCAA
	GAGGCTTCGTACAGCTTGGT
Fusarium culmorum mitovirus 1	AGAATCAGCGGGACCAAACG
	TCGAATGTTCCGTCCTGAGG
Fusarium culmorum mitovirus 2	ACAGAGGTCCCCGTTAGCTT
	AATGTCGCGTCACAGGTCAT
